# Antimicrobial resistance profiles of *Escherichia coli* derived from an integrated agroforestry–livestock system in Deli Serdang Regency, Indonesia

**DOI:** 10.14202/vetworld.2024.690-699

**Published:** 2024-03-22

**Authors:** Rita Rosmala Dewi, Arif Nuryawan, Saleh Mohammed Jajere, Juli Mutiara Sihombing, Ika Julianti Tambunan

**Affiliations:** 1 Department of Animal Husbandry, Faculty of Science and Technology, Universitas Tjut Nyak Dhien, Medan, Indonesia; 2 Department of Forestry, Faculty of Forestry, Universitas Sumatera Utara, Medan, Indonesia; 3 Department of Veterinary Public Health and Preventive Medicine, Faculty of Veterinary Medicine, University of Maiduguri, Maiduguri, Borno State, Nigeria; 4 Department of Pharmacy, Faculty of Pharmacy, Universitas Tjut Nyak Dhien, Medan, Indonesia

**Keywords:** agro-silvopastoral, antimicrobial Resistance, *Escherichia coli*,Indonesia, livestock, multidrug resistance

## Abstract

**Background and Aim:**

Antimicrobial resistance (AMR) has become a significant global concern. Epidemiological data do not provide a robust description of the potential risks associated with AMR in the integrated agroforestry–livestock systems in Indonesia. Thus, the present study investigated the phenotypic and multidrug resistance (MDR) profiles of *Escherichia coli* strains isolated from the feces of livestock raised in the agro-silvopastoral system in Deli Serdang Regency, North Sumatra Province.

**Materials and Methods:**

A standard microbiological culture procedure was followed to isolate the organism and test antibiotic susceptibility using the Kirby-Bauer disk diffusion protocol. Furthermore, the multiple antibiotic resistance index was determined. Univariate analysis was conducted to identify the risk factors associated with AMR.

**Results:**

The vast majority (77.5%) of livestock farmers were aged >30 years. All farmers were men and had no higher education (100% of them). The majority of the animal species managed were cattle and goats (37.5% each) and the livestock grazing pasture system (67.5%). In addition, the majority of farmers reported high antimicrobial use on their farms (87.5%). Of the samples (n = 142) analyzed, n = 70 were positive, with an overall prevalence of 44.4%. The species-specific prevalences of *E. coli* were 32.5%, 47.8%, and 50% in buffalo, goat, and cattle, respectively. Ampicillin and tetracyclines exhibited high resistance levels among the studied animal species. A relatively lower MDR for *E. coli* was associated with grazing on the pasture.

**Conclusion:**

The findings from the current study provide baseline epidemiological information for future robust studies aimed at elucidating the drivers and patterns of AMR in agro-silvopastoral systems in the study area or elsewhere.

## Introduction

Because of the rapid growth of intensive animal production systems in response to the global demand for animal protein, concerns have arisen regarding the potential emergence of antimicrobial resistance (AMR). This concern stems from the frequent use of antimicrobial agents in these systems to maintain animal health and enhance productivity [1]. Antimicrobials are commonly used to treat, manage, and prevent infectious diseases in animal feed. Antimicrobials are also employed for non-therapeutic purposes, such as improving feed efficiency [2, 3], in several countries worldwide. Owing to the current global surge in antimicrobial use, it has been estimated that antimicrobial use will expand to approximately 11.5%, reaching a total of 104,079 tons across the globe by 2030 [4]. The Asian region has been identified as a “hotspot” as a predominant “antimicrobial consumption cluster” accounting for 67% of global antimicrobial consumption. Notably, Southeast Asian countries, such as Indonesia, Thailand, and Vietnam, have recently reported an enormous increase in antimicrobial use patterns in livestock [5].

Antibiotics are becoming increasingly ineffective and pose one of the greatest threats to humans, animals, and the environment from a one health perspective [6]. One of the major drivers and contributing factors for the emergence and spread of AMR in livestock production settings is the frequent use of these agents either for prophylactic (as feed additives to promote growth performance) or therapeutic purposes (treating bacterial infections) [7]. If pathogenic organisms develop resistance to a particular antimicrobial, the agent will no longer inhibit their growth, resulting in treatment failure and high mortality rates. Thus, the development of AMR and multidrug resistance (MDR) causes high medical care costs, longer hospital stays, and increased mortality rates in humans [8, 9]. Similarly, AMR has negatively impacted livestock farmers through treatment failures, production losses, and economic losses, thereby posing potential risks to the overall viability of the animal sub-sector [10]. Moreover, the incidence of AMR in animal health is increasing because of its involvement in diverse animal species and microbes, varying livestock environments, and intricate resistance mechanisms [11, 12]. Previous studies have identified livestock, such as ruminants, as plausible reservoirs of antimicrobial-resistant bacteria and resistance genes, including *Escherichia coli* [13-15].

*E. coli* has been frequently used for monitoring AMR in cattle and animal-derived foods because it is ubiquitous and predilects the digestive tract of warm-blooded animals [16]. Although some significant strains of *E. coli* are known to establish commensal relationships with their bovine hosts [17], others are considered the most common cause of diarrhea in cattle [18]. During the past decades, there has been an increasing trend of antimicrobial-resistant *E. coli* with an increasing frequency of resistance genes, many of which were acquired through horizontal gene transfer – posing a significant threat to livestock, humans, and the environment [19]. This trait may accelerate the emergence of AMR in *E. coli* from livestock and the environment, including in animal production settings in integrated agroecosystems [20]. Integrated systems, such as livestock agroforestry, are frequently adopted to manage cattle production [21]. In Indonesia, livestock farming in the agro-silvopastoral system, a land management approach that integrates agricultural, forestry, and animal husbandry practices, is a government initiative to promote sustainable forest land management and enhance food security [22, 23].

A sustainable system can benefit local communities close to forests while promoting sustainable food security. Therefore, the presence of AMR and MDR *E. coli* in the integrated system is a concern because *E. coli* is also a major reservoir of resistance genes that may be responsible for the emergence of MDR in the system. Manyi-Loh *et al*. [24] examined the development and emergence of AMR and MDR organisms in integrated agroecosystems. Furthermore, a study conducted in Ethiopia revealed that AMR and MDR *E. coli* circulate in livestock and silvopastoral settings [25]. As a result, the potential threat of MDR commensal *E. coli* from livestock has become a recent concern that should be monitored in integrated livestock agroforestry.

Therefore, this study aimed to investigate the AMR patterns and MDR of *E. coli* isolated from ruminant livestock manures in an integrated livestock–agroforestry system in Deli Serdang Regency, North Sumatra, Indonesia.

## Materials and Methods

### Ethical approval and Informed consent

Ethical approval was not required for the current investigation. This was because only fecal samples (freshly voided on the farm surface) were collected without any contact with the animals or harm to them. In addition, the current investigation is not an experimental study and does not involve any invasive procedure (collecting tissue or blood samples) on livestock. Finally, livestock farmers were briefed on the scope of the study, after which written informed consent was obtained.

### Study period and location

This study was conducted from May 2023 to October 2023 in Deli Serdang Regency, North Sumatra, Indonesia. The study population comprised ruminant livestock raised in an integrated livestock–agroforestry system in Deli Serdang Regency, specifically in four districts: Pancur Batu, Sunggal, Pantai Labu, and Batang Kuis. The target population comprised eight extensive livestock farms that were selected using convenience non-probability sampling [26]. As this sampling procedure may introduce bias on the outcomes (parameter estimates) of the study and thus limit its generalizability, the researchers attempted to reduce this bias by modifying this sampling technique using the following steps as described elsewhere [27, 28]: First, we diversified our data collection procedure by collecting farmers from four different districts, namely Pancur Batu, Sunggal, Batang Kuis, and Pantai Labu. Second, using an established method, we employed a sample size calculation to determine the required sample size. Finally, multiple sources were used to ensure that the drawn samples were representative of the large population. The farmers were selected on the basis of their willingness to participate in this study. Moreover, the farms included were obtained through multiple recruitment processes, such as officer recommendations or in-person interviews.

### Sample size determination

We calculated the sample size using the formula described by Thrusfield [29]: Assuming a 95% confidence interval (CI), an absolute precision of 5%, and an expected prevalence of *E. coli* of 8.6%, as previously reported in the feces of Indonesian cattle [30], the required sample size was determined as n = 126. However, the sample size was increased to n = 142 to increase the precision and counter for damaged samples during sample processing.

### Structure of the questionnaire and sample collection

The questionnaire consisted of open-ended questions on demographic data, farm hygiene, antibiotics used on farms, other medicines used on farms, veterinarian support, and animal species raised on farms. Before administering the questionnaire, the farmers were verbally informed of the scope of the study, and consent was obtained from them after agreeing to participate in the study. However, farmers who refused to participate or did not provide consent were excluded from the study. A total of 142 fresh fecal droppings from all the animals included in the present study were collected from the ground a few seconds or minutes after deposition using an aseptic technique and subsequently stored in sterile polyethylene bags for preservation until transport and processing in the laboratory. For further bacteriological analysis, samples were placed in cool boxes equipped with ice packs and transported to the microbiology laboratory of the Faculty of Pharmacy, Tjut Nyak Dhien University.

### Bacterial isolation and identification

Methods for *E. coli* isolation and identification were conducted in accordance with established protocols [25, 31]. Briefly, 2 g of the livestock fecal sample was mixed with 18 mL of buffer phosphate water and incubated at 37°C for 24 h. Subsequently, a loop-full of pre-enriched cultures was taken, inoculated on eosin-methylene-blue (EMB) agar, and incubated at 37°C for 24 h. Colonies exhibiting a metallic sheen on incubated EMB agar were identified as *E. coli*-positive. We subsequently cultured the samples on nutrient agar for further confirmation using additional biochemical tests, including triple sugar iron agar, urease, indole, methyl red, Voges-Proskauer, and citrate tests.

### Antimicrobial susceptibility test

Antimicrobial susceptibility was determined on Mueller-Hinton agar using the Kirby-Bauer disk diffusion method, following the guidelines provided by the Clinical and Laboratory Standard Institute [32]. Antibiotic selection was determined according to the WHO and OIE recommendations for antimicrobial use in humans and food-producing animals [33, 34]. This selection was aligned with the integrated AMR surveillance strategy in Indonesia [35]. A panel of five antibiotic disks, including ampicillin (AMP) (10 μg), gentamicin (10 μg), tetracycline (TE) (30 μg), ciprofloxacin (CIP) (5 μg), and chloramphenicol (CHL) (30 μg), was used. Resistance was defined as any isolate that exhibited resistance to one or more of the analyzed agents. In addition, isolates demonstrating resistance to the three classes of antimicrobials are referred to as MDR. The Multiple Antibiotic Resistance (MAR) index was calculated by dividing the number of antibiotic types to which a certain isolate exhibited resistance by the total number of antibiotics to which the isolate was exposed [36].

MAR index = *a/b*, where “a” represents the number of antibiotics to which the isolates demonstrated resistance and “b” represents the total number of antibiotics to which the isolate was subjected [36].

### Statistical analysis

Data were first imported into Microsoft Excel 2019 (Microsoft Corporation, New York, USA). We descriptively analyzed the data by calculating the prevalence of *E. coli*. Figures and frequencies were also obtained. Antimicrobial susceptibility test data for *E. coli* isolates were determined using WHONET 5.6 [37, 38]. We recorded and compared the MDR prevalences of *E. coli* isolated from the studied animal species. Further statistical analyses of the data were conducted using SPSS version 26.0 (IBM Corp., Armonk, NY, USA). p < 0.05 (α = 5%) was considered significant.

## Results

### Demographic characteristics of the farms

Agroforestry refers to the integration of the cultivation of forestry plants with agricultural crops and/or livestock [39, 40]. The current definition used by the International Center for Research in Agroforestry involves a comprehensive categorization of land-use systems and practices aimed at enhancing overall land productivity [40]. This involves the simultaneous or sequential cultivation of agricultural, forest, and/or livestock crops on the same land unit while adhering to local traditional management practices [40, 41]. Agro-silvopastoral systems integrate the production of animals with trees and crops [42, 43]. In North Sumatra Province [44, 45], including Deli Serdang Regency [46, 47], livestock production integrated with oil palms and forests has been reported. Crops and oil palm plantations predominate on agricultural land in this area, whereas goats, cattle, broilers, and laying chickens and pigs are the main animal producers [48].

According to the Statistic Central Bureau (BPS) of North Sumatra, Deli Serdang regency contributes approximately 13% cattle, 18% goat, 16% sheep, and 2% buffalo to the total animal production in North Sumatra [49]. In this area, agriculture and animal husbandry have a huge potential to provide sustainable food and meet the basic needs of the local population. According to the BPS, growth and production data in Deli Serdang Regency have recently experienced an increasing trend. For example, data on beef production showed an increase of 4,595,593 kg in 2021 compared with 4,376,778 kg recorded in 2020 [49]. The study involved eight farmers from four districts: Pancur Batu (n = 2), Sunggal (n = 2), Pantai Labu (n = 2), and Batang Kuis (n = 2). Ruminant livestock raised in this system were goats (n = 3; 37.5%), cattle (n = 3; 37.5%), and Murrah buffalo (n = 2; 25%).

### Descriptive statistics of the farms

Most farmers in the agro-silvopastoral areas were male (n = 8; 100%) and above 30 years of age (87.5%). The majority of farmers did not attend university (n = 8; 100%). Cattle constituted 50% (n = 4) of the area’s farming, whereas goats (n = 2) and buffaloes (n = 2) made up the remaining 50%. Nearly all farm owners (n = 6; 75%) implemented sanitation, and approximately one-quarter of them received support from veterinarians. More than half of the farm owners (n = 5; 62.5%) rely on pasture-based systems for livestock grazing, whereas the remaining 37.5% (n = 3) incorporate forest forage into their cattle diet.

### Use of antibiotics in livestock

Table-1 presents the antimicrobial usage patterns of farmers in agro-silvopastoral settings. Antimicrobial usage was relatively high (n = 7; 87.5%), and farmers predominantly used antibiotics when the animals were sick (87.5%). Among the participants involved in the administration of antibiotics to livestock, 37.5% relied on animal health officials, whereas 62.5% engaged in self-prescription. All farmers involved in this system reported the use of oxytetracycline as a single antimicrobial in animals.

**Table-1:** Farmer’s sociodemographic characteristics, livestock management practices, and antimicrobial usage.

Variables	Frequency (%)
Farmer sociodemographic information	
Age	
<30 years	1 (12.5)
>30 years	7 (87.5)
Gender	
Male	8 (100)
Female	0 (0)
Formal education	
Non-higher education	8 (100)
Higher education	0 (100)
Districts	
Pancur Batu	2 (25)
Pantai Labu	2 (25)
Sunggal	2 (25)
Batang Kuis	2 (25)
Livestock management	
Species	
Cattle	3 (37.5)
Goat	3 (37.5)
Buffalo	2 (25)
Sanitation	
Hygiene application	6 (75)
No Hygiene application	2 (25)
Animal health official support	
Yes	2 (25)
No	6 (75)
Grazing of animals on pastures	
Yes	5 (67.5)
No	3 (37.5)
Antibiotic application	
Antibiotic usage	
Yes	7 (87.5)
No	1 (12.5)
Treatment	
Yes	8 (100)
No	0 (0)
Preventive	
Yes	8 (0)
No	0 (100)
Based on animal health official prescribe	
Yes	5 (37.5)
No	3 (62.5)
Drugs application (antiparasitic)	
Yes	4 (50)
No	4 (50)

### Prevalence of *E. coli*

In the four districts that make up the Deli Serdang regency, the frequency of recovery of *E. coli* in livestock raised in agro-silvopastoral settings was 44.37% (95% CI: 36.4–52.9). Specifically, the prevalences in cattle, goats, and buffalo were 50% (95% CI: 36.3–63.6), 47.8% (95% CI: 32.9–63.1), and 32.5% (95% CI: 18.6–19.1), respectively.

### AMR patterns

**Figure-1:**
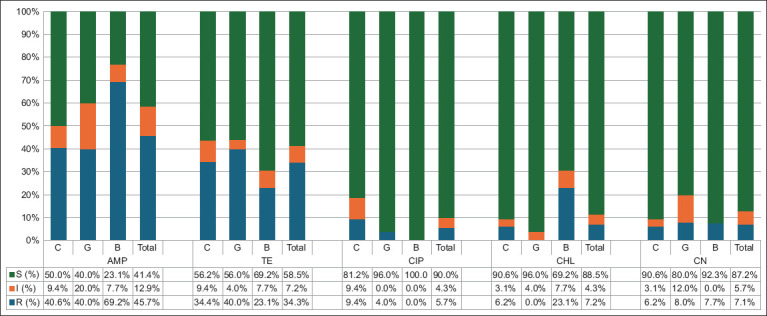
The prevalence of antibiotic resistance in *E. coli* isolates (n = 70) observed on livestock raised in the agro-silvopastural system in Deli Serdang Regency. C=Cattle, G=Goat, B=Buffalo, AMP=Ampicillin, TE=Tetracycline, CIP=Ciprofloxacin, CHL=Chloramphenicol, CN=Gentamycin, R=Resistant, I=Intermediate, S=Susceptible, *E. coli=Escherichia* *coli*.

Figure-1 presents the AMR profiles of n = 70 *E. coli* isolates. In general, *E. coli* isolates obtained from ruminants cultivated in the agro-silvopastoral system demonstrated high resistance to AMP (45.7%; 95% CI, 33.7–58.1) and TE (34.3%; 95% CI, 23.4–46.6). Lower levels of resistance to CHL (7.2%; 95% CI = 2.4–15.9), gentamicin (7.1%; 95% CI = 2.4–15.9), and CIP (5.7%; 95% CI = 1.6–13.9) were documented in the isolates.

Figure-1 illustrates the resistance levels of *E. coli* isolates recovered from different livestock species. In general, cattle isolates demonstrated high resistance to AMP (40.6%; 95% CI = 24.2%–59.2%) and TE (34.4%; 95% CI = 19.2%–53.2%), whereas CIP (6.2%; 95% CI = 2.5–26.2%) exhibited lower resistance. Similarly, high resistance levels to AMP and TE were recorded in buffalo (69.2%; 95% CI = 38.9–89.6 vs 23.1%; 95% CI = 6.2–54.0) and goats (40%; 95% CI = 21.8–61.1 vs. 40%; 95% CI = 21.8–61.1). However, CHL and CIP are effective against the *E. coli* strain recovered from goats and buffaloes, respectively.

Table-2 presents the AMR characteristics of *E. coli* isolates derived from different animal species. A high proportion of AMP (40%–69.2%) and TE (23%–40%) resistant *E. coli* isolates were observed in cattle, goats, and buffaloes. In general, *E. coli* isolates obtained from Bs were more resistant to AMP and CHL than those obtained from buffaloes. In addition, *E. coli* strains isolated from goats and cattle showed a higher percentage of resistance to TE and CIP. CHL resistance exhibited by *E. coli* isolates was statistically associated with the number of examined animal species (p < 0.05) (Table-2).

**Table-2:** Antimicrobial resistance patterns of *E. coli* isolates recovered from Cattle, Goat, and Buffalo raised in agro-silvopastoral system of Deli Serdang regency, North Sumatra.

Antibiotics	Resistance (%)			Chi- square	p-value

	Cattle	Goat	Buffalo		
Ampicillin	40.6	40	69.2	2.181	0.336
Tetracycline	34.4	40	23.1	0.748	0.688
Ciprofloxacin	9.4	4	0	3.917	0.141
Chloramphenicol	6.2	0	23.1	6.300	0.043*
Gentamycin	6.2	8.0	7.7	1.794	0.408

* Statistically significant, *p* < 0.05, *E. coli*=*Escherichia coli*

### MDR

**Figure-2:**
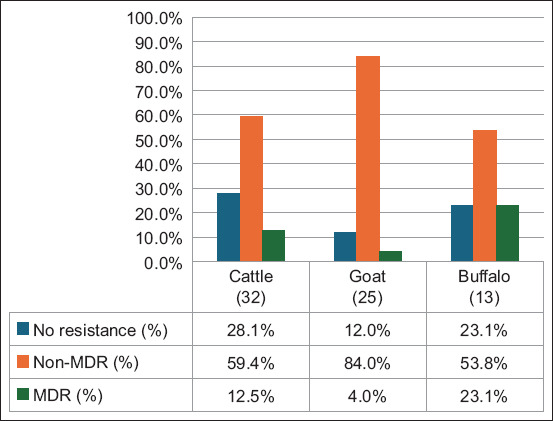
MDR of *E. coli* recovered from livestock increased in the agro-silvopastural system in Deli Serdang District of North Sumatra. Numbers inside the brackets “()” denote the number of isolates; those on bars indicate percent isolates showing resistance; non-MDR = only 1 or 2 classes; MDR=Multidrug resistance. *E. coli=Escherichia* *coli*.

As depicted in Figure-2, the prevalences of MDR among *E. coli* isolates recovered from buffalo, cattle, and goats were 23.1% (95% CI = 5.1–53.8), 12.5% (95% CI = 3.5–28.9), and 4% (95% CI = 1.0–20), respectively. *E. coli* isolates from buffaloes and cattle had significantly higher MDR levels, whereas isolates from goats had significantly lower MDR levels.

Univariate analysis revealed that ruminant livestock in Deli Serdang Regency, North Sumatra grazing on pastures (p = 0.045) was significantly associated with the presence of MDR *E. coli* in the agro-silvopastoral system. As shown in Table-3, this association was observed in integrated system settings in the aforementioned region of Indonesia.

**Table-3:** Univariable analysis of risk factors associated with the occurrence of MDR *E. coli* recovered from livestock raised in the agro-silvopastoral system of Deli Serdang Regency, North Sumatra, Indonesia.

Variables	Frequency	Positive (%)	Chi-square	p-value
Species				
Cattle	32	4 (12.5)	3.142	0.208
Goat	25	1 (4)		
Buffalo	13	3 (23.1)		
Sanitation				
Yes	56	5 (8.9)	1.729	0.189
No	14	3 (21.4)		
Animal health official support				
Yes	14	3 (21.4)	1.729	0.189
No	56	5 (8.9)		
Grazing of animals on pastures				
Yes	38	7 (18.4)	4.015	0.045*
No	32	1 (3.1)		
Antibiotic application				
Yes	61	8 (100)	1.333	0.248
No	9	0 (0)		
Animal health official prescribe				
Yes	24	3 (12.5)	0.041	0.839
No	46	5 (10.9)		

* Statistically significant, *p*< 0.05, *E. coli*=*Escherichia coli*

### MAR Analysis

Table-4 presents the MAR indices of the *E. coli* isolates. Of the 70 *E. coli* isolates tested, 14 (20%) were susceptible to all tested antibiotics, whereas 24 (34.3%) were resistant to at least one antibiotic. Furthermore, 21 isolates (30%) exhibited resistance to two antibiotics, 7 isolates (10%) showed resistance to three antibiotics, and 1 isolate (1.4%) showed resistance to four antibiotics (Table-4). The most common co-resistant phenotype was AMP and TE resistance in 27.5% of the isolates tested. The MAR index ranged from 0 to 0.8, with an average MAR index of 0.27. Of the isolates tested, isolates originating from buffalo had an average higher MAR value (0.3). In addition, goat isolates had the lowest average MAR value of 0.2.

**Table-4:** MAR of *E. coli* isolates.

Number of antimicrobials resistant	Predominant antibiotic resistance profile	MAR Index	No. of Isolates (%)
0	-	0	14 (20)
1	Amp; Tet; Chl; Gen	0.2	24 (34.3)
2	AmpTet; AmpGen; AmpChl; AmpCip; CipTet; GenTet	0.4	21 (30)
3	AmpCipTet; AmpGenTet; AmpChlTet	0.6	7 (10)
4	Amp Gen Chl Tet	0.8	1 (1.4)

MAR=Multiple antibiotic resistance index, Amp=Ampicillin, Cip=Ciprofloxacin, Chl=Chloramphenicol, Te=Tetracycline, *E. coli*=*Escherichia coli*

## Discussion

The emergence of AMR in livestock is gaining attention because of its significant consequences on animal and human health [50]. While much attention has been focused on animal species raised in typical farm production settings, AMR in livestock from integrated livestock and agroforestry systems may also serve as a comparable source of AMR risks to animals, humans, and the environment (One Health approach). AMR in *E. coli* derived from ruminant livestock reared within this particular system, which is widely implemented across several regions of Indonesia [44–46, 51], is limited. In the current study, most livestock farmers reported high antimicrobial usage on their farms. The prevalence of *E. coli* was 44.4% (n = 70, 95% CI: 36.4–52.9), and species-specific prevalence varied across the studied animal species, ranging from 32.5%–50%. The recovered *E. coli* isolates exhibited high resistance to AMP and TE. However, lower MDR levels were observed in isolates associated with pasture grazing.

*E. coli* isolates derived from cattle exhibited high resistance to AMP and low resistance to CHL and gentamicin. The prevalence of AMP (49%) and TE resistance (23%) observed in cattle production in the forest interface system aligned with the findings reported previously [52]. In addition, a study [25] reported significantly high resistance levels against TEs in isolates collected from cattle raised in the silvopastoral system of Ethiopia. Consistent with the findings of the current study, *E. coli* isolates from the cattle production systems of Zimbabwe (78%), Ghana (54.8%), Mexico (68.8%), and Indonesia (35.5%) demonstrated similarly high levels of resistance against AMP [53–56]. It should be noted, however, that this tendency appears to be more pronounced than the findings of this study. This observed lower tendency may be attributed to the fact that most cattle production practices within the agro-silvopastoral system consist of small-scale production systems, which may involve less frequent use of medications (e.g., antimicrobials) compared with intensive cattle production [55].

AMP and TE had the highest proportion of resistant isolates of *E. coli* from goats, whereas CHL had the highest susceptibility. This finding is not comparable with that of goats raised in the silvopastoral system, where the level of resistance to TE was lower (13.6%) than in the present study [25]. Notwithstanding, our findings are consistent with those of a similar study conducted in Nigeria, where *E. coli* isolates from goat feces exhibited high resistance levels to AMP (94.7%) and TE (89.5%) [57]. However, the same study also reported high CHL resistance (68.4%). A similar study conducted in Qatar reported relatively high resistance of *E. coli* isolates recovered from the feces of healthy goats to TEs (34%) [58]. In contrast, Manishimwe *et al*. [59] and Srivani *et al*. [60] reported lower levels of resistance to AMP and TE, with proportions of 7.4% and 14.1%, respectively. In addition, *E. coli* resistance to CIP (4.7%) has been reported [60]. In a similar study conducted in Italy, lower resistance to AMP (46.8%) and higher resistance to TE (34.0%) and gentamicin (8.5%) were observed [61]. The observed discrepancies in the aforementioned outcomes might be attributed to the use of distinct approaches for sampling and isolation techniques, age of the livestock, demographics, health status of the livestock, intensity of antimicrobial usage, and exposure levels of the livestock to different classes of antibiotics [62].

To the best of our knowledge, this study is the first to explore the AMR profiles of *E. coli* derived from the feces of buffaloes in North Sumatra, Indonesia. In general, *E. coli* isolates derived from buffaloes demonstrated slightly higher resistance levels to AMP and CHL than those from cattle and goats. A statistically significant difference was observed in the AMR of *E. coli* isolates against CHL across animal species. This finding was unexpected, particularly because none of the buffalo farms involved in this study reported CHL use. In addition, the use of CHL in livestock is prohibited in accordance with the regulations set by the Indonesian government [63]. A plausible reason could be that the development of AMR by pathogens is a complex phenomenon influenced by multiple factors that may not be linked to the exposure or use of specific antimicrobial agents in livestock farming settings [64]. Moreover, there is evidence that some AMR genes undergo co-selection or exhibit genetic linkages [65, 66]. This phenomenon entails the genetic association between resistance to one antimicrobial agent and resistance to another, resulting in joint vertical or horizontal transfer among bacteria of the same or different species [64]. The CHL resistance patterns observed in this integrated system may require further scientific investigation into the epidemiology and dynamics of the resistance mechanisms and their spread.

These results indicate high TE and AMP resistance across animal species and districts. One possible explanation for this is the widespread use of these drugs in the local livestock industry. According to Yusuf *et al*. [67], TE is extensively used as an antibiotic in the cattle industry in Indonesia. Furthermore, TE and AMP have been widely used to treat infections in humans and animals [68]. TE is also widely used globally as a growth enhancer, commonly at subtherapeutic levels [69].

A high proportion (75.7%) of the *E. coli* isolates in the current study had MAR indexes between 0.2 and 0.8, indicating resistance to at least one or more antibiotic agents. This may be attributable to the high use of antibiotics in the farms. In general, samples with a MAR index >0.2 are associated with a high risk of contamination [25, 36]. This may indicate an indiscriminate use of antibiotics in the agro-silvopastoral environments of the farms studied or even between agriculture and humans in the ecosystem. The interlinked nature of the soil, plants, and animals within this integrated system establishes a robust correlation, suggesting a suitable pathway for the emergence and dissemination of AMR and AMR genes in the agroforestry system [24]. Antimicrobial dissemination can occur in all of these pathways, establishing a complex network through which AMR genes can be transmitted [70]. Manure waste from livestock production settings and its subsequent application to agriculture as a source of essential nutrients needed for crop production may serve as a direct link between antibiotics, animal production settings, and the environment in integrated production systems [20]. A study conducted in Ethiopia showed that soils contaminated with livestock faces might serve as an important source of drug-resistant *E. coli* to livestock raised in integrated production systems [25].

The global emergence of MDR organisms has been recognized [71, 72]. In the current investigation, the MDR level of *E. coli* in cattle was 12.5%, which was comparatively lower than the reported MDR level of 26.7% in Ethiopia [25] and much higher than that reported in studies from Mexico (72.7%) [54] and Ghana (64.3%) [56]. Similarly, the MDR level of *E. coli* in the current study was also lower than that of goats from Qatar (44%) [58] and Rwanda (13.1%) [59]. Limited reports on MDR in *E. coli* from buffaloes have suggested very high MDR levels (69.8%) [60] compared with the results of this study. Although the current study recorded lower MDR *E coli* across the studied animal species compared with those reported in livestock production systems [25, 58–60], the agro-silvopastoral system serves as a possible route for the emergence and spread of MDR. These complex nexuses and linkages among livestock, crops, and the environment present an increased likelihood of expediting the emergence and spread of AMR through animal manure sources [20]. Therefore, it is important to consider the possible routes to AMR emergence and spread. Manure is a substantial source of AMR and AMR genes [73]. Manure from livestock may serve as an excellent route through which antimicrobial-resistant bacteria and AMR genes can enter the environmental soil and water systems [66, 74]. Furthermore, commensal *E*. *coli* strains can transfer acquired resistance traits to pathogens such as *Salmonella* or pathogenic *E*. *coli* through horizontal gene exchange, which is primarily facilitated by conjugation, enabling the transfer of AMR genes between different bacterial populations [75, 76]. Therefore, this scenario could accelerate the dissemination of MDR through the ecosystem, which would jeopardize public health.

Animal grazing on pastures was significantly associated with MDR *E. coli* recovered from the agro-silvopastoral system. According to EFSA [77], systems with outdoor access and grazing are more likely to be influenced by external environmental sources of AMR bacteria and AMR genes related to grazing than those maintained indoors. Markland *et al*. [78] suggested that soil, plants, and water are critical sources of AMR pathogens in grazing cattle. A high prevalence of AMR bacteria may occur during grazing or foraging activities where cattle inadvertently ingest soil, plants, or forages contaminated with AMR bacteria. In addition, grazing animals have a high probability of contact with other animal species or wildlife interfaces in livestock–agroforestry systems, which could accelerate the risk of AMR emergence and spread. In a study from South Africa [79], AMR bacteria and genes were exchanged between livestock and wildlife co-grazing in the same environment. This exchange occurs because, during co-grazing, direct interspecies contact may occur during the sharing of pastures or water points or indirectly through mobile transfer vectors, such as wild birds or wind [80].

## Conclusion

To the best of our knowledge, this is the first report of AMR and MDR *E. coli* recovered from an integrated livestock–agroforestry system in North Sumatra, Indonesia. Only a few samples, which may not represent the general population, were analyzed in the current study because of limited funding. Therefore, interpretation and generalization of the findings should be performed with caution. Despite these limitations, the findings provide valuable insights into the status of AMR and MDR *E. coli* derived from ruminant livestock, as well as associated risk factors in the agro-silvopastoral systems in the North Sumatra region, Indonesia. In general, the resistance levels demonstrated by the *E. coli* isolates across the studied animal species were low, with the highest detected against AMP and TE. Although a lower MDR level of *E. coli* was reported in this study, it may play an important role in the expansion of MDR in the ecosystem. Furthermore, grazing animal management in the integrated system was found to be significantly associated with the occurrence of MDR *E. coli*, suggesting a possible role played by the environment in the dissemination and spread of AMR and AMR genes. Therefore, it is recommended to continue monitoring and intensively investigating AMR in integrated livestock and agroforestry systems, which will enable the formulation of well-informed strategies and approaches to effectively tackle the challenges of AMR in the future.

## Authors’ Contributions

RRD, AN, and SMJ: Conceptualization, methodology, extracted, verified, and analyzed the data, and drafted and revised the manuscript. JMS and IJT: Data curation and fieldwork. All authors have read, reviewed, and approved the final manuscript.
